# Man versus Machine versus Ribozyme

**DOI:** 10.1371/journal.pbio.0060132

**Published:** 2008-05-27

**Authors:** Andrew D Ellington

## Abstract

A microfluidic device has been constructed to carry out the automated, continuous evolution of ribozymes. A comparison with manual efforts reveals that both are capable of far flung forays into sequence space.

*The steam drill was on the right hand side,*

*John Henry was on the left, *

*Says before I let this steam drill beat me down, *

*I'll hammer myself to death”*—The Ballad of John Henry (American, traditional)

Organisms and molecules achieve evolutionary success via many possible paths, what evolutionary biologists sometimes call the “tempo and mode” of evolution [1]. In contrast, engineered machines do not typically adapt to new functions during their normal operation. This seemingly inherent difference between the vicissitudes of living things and the exactitudes of mechanisms has frequently fascinated writers. One famous folktale of the Americas, the legend of John Henry, pits the evolved strength of a railroad worker, a “steel-driving man,” against the inhuman strength of a steam-powered rail driver. In a recent issue of *PLoS Biology*, a similar competition improbably played out again, with an RNA enzyme as the backdrop.

Experimentalists can carry out a wide range of in vitro evolution experiments that “force” the selection of molecules with particular phenotypes. One of the greatest successes of in vitro evolution to date was the evolution of ribozyme ligases from a random sequence population by David Bartel and Jack Szostak [2]. These authors asked a pool that spanned 220 random sequence positions to ligate an oligonucleotide to itself (Figure 1A). Those ribozymes that could do so would gain access to a covalently linked primer-binding site, and thus to replication via reverse transcription and PCR amplification. After multiple cycles of selection and amplification, a number of ligases indeed emerged from the population, having outperformed their nonligase (and nonreplicable) competitors. One of the ligases was both extremely complex and extremely fast, for a ribozyme (*k*_cat_ of 100 min^−1^, upon optimization) [3]. While evolution had obviously occurred, it was very different from biological evolution: the original population was exceedingly complex (containing upwards of 10^15^ different variants) and was progressively winnowed through a selection process in which enzymatic function and sequence amplification were separated from one another in both time and space.

**Figure 1:**
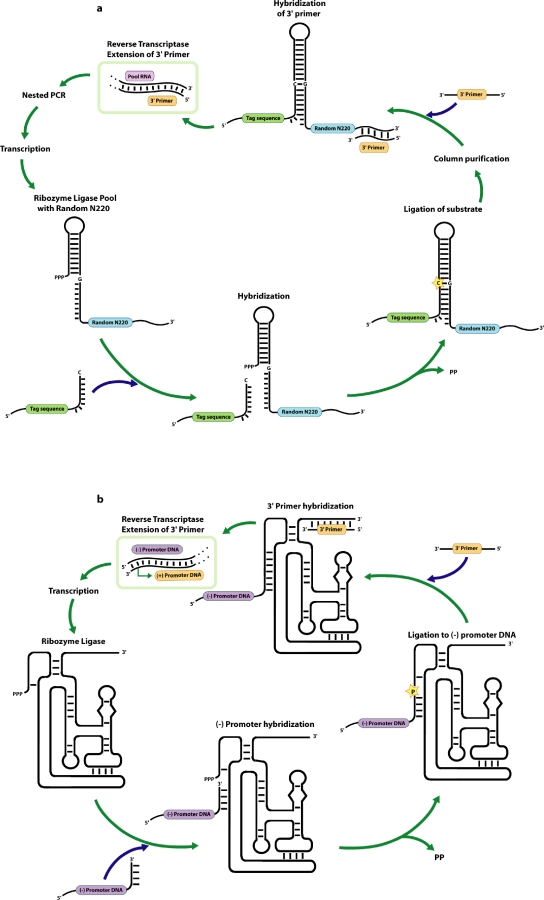
Discontinuous and Continuous Selection of a Ribozyme Ligase (A) Discontinuous selection of the Bartel class I ligase. A random sequence pool was incubated with a short constant sequence oligonucleotide. Those members of the population that performed a ligation reaction could be subsequently amplified by reverse transcription, PCR, and in vitro transcription. (B) Continuous evolution of the Bartel ligase. The same ligation reaction takes place, but in this instance, the oligonucleotide attaches and completes a T7 RNA polymerase promoter. Upon reverse transcription, the promoter is rendered double-stranded and thus can be used immediately for transcription of the adjacent ribozyme template. This allows ligation, reverse transcription, and transcription to occur side-by-side in the same reaction mixture, and thus for replication and evolution to occur continuously.

The so-called Bartel ligase was further evolved for catalytic efficiency by Martin Wright and Jerry Joyce [4]. These authors made a very clever adaptation to the original ligase in which a functional promoter was formed only upon ligation of the oligonucleotide substrate to the ribozyme (Figure 1B). This adaptation allowed enzymatic function and sequence amplification to be coupled at the same time and in the same test tube. In this regard, evolution now occurred almost continuously, just as in the wild, at least until the test tube ran out of “food” (substrates) for ribozyme replication. To circumvent this problem, Wright merely had to regularly transfer a portion of the reaction to a new food source. While the adaptations necessary for continuous evolution severely depressed the reactivity of the ribozyme over generations of so-called continuous evolution, the Bartel ligase accumulated multiple mutations and became almost as efficient as its parent (*k*_cat_/*K*_m_ of 1 × 10^7^ M^−1^ min^−1^, an improvement of >10^4^-fold). This continuous evolution was more akin to what normally occurs in biology, and while some mutations were likely fixed from an initial, heavily mutagenized (8% per position) population, others clearly arose during the experiment itself.

The 1,000 manually guided doublings of the ligase that were shepherded to fruition by the labors of Wright were apparently not enough to satisfy management. Paegel and Joyce [5] now describe a machine that can continuously evolve the ligase. In their microfluidic device, new food is fed to the rampantly replicating population not by hand, but by a series of valves controlled by a computer. As ligated ribozymes accumulate, they intercalate a dye—thiazole orange—in the reaction mixture, and this in turn gives off a fluorescent signal that can be seen by embedded sensors. Once fluorescence reaches a given level, new food flows, the ribozymes (and fluorescence) are diluted, and replication continues until fluorescence again builds and the food gates are again opened. In these experiments, the ribozymes were under selection for speed, but also for their ability to hold onto their oligonucleotide substrate. The *K*_m_ of the starting ribozyme was 35 μM, and substrate concentration was decreased from a limiting 1 μM to as low as 0.05 μM over the course of the continuous evolution experiment. As before with the manual regime the ribozyme responded, fixing multiple mutations that resulted in an improvement in its *K*_m_ to 0.4 μM. Interestingly, no real improvement in speed (*k*_cat_) was observed. As before, while some mutations may have arisen from the original (a much more modest 0.7% per position) population, sequencing of intermediate variants during the selection suggested that a number of mutations arose during the course of the experiment. Some of these mutations were clearly interdependent, and could only have conferred a selective advantage in the context of previously fixed mutations. The evolution in the microfluidic device is increasingly similar to what might happen in the wild, or at least in a feedlot, with ribozymes constantly fed and allowed to fight for resources with no temporal delay or human intervention.

A summary of the outcomes of continuous but manual evolution, and continuous automated evolution can be seen in Table 1. In directly comparing these results, it should be kept in mind that Wright started with one variant of the Bartel ligase (b1-207) that had to be adapted to continuous evolution, while Paegel started with a variant (B16–19) that had been pre-adapted in a different continuous, manual evolution experiment [6]. Nonetheless, other variables can be more directly compared: both continuously evolved ribozymes appear to have topped out in terms of speed, and ultimately obtained similar *K*_m_ values. This latter observation is surprising, given that the machine-based selection was heavily weighted towards improving *K*_m_, and Paegel and Joyce suggest that an additional 20-fold improvement in *K*_m_ was theoretically possible.

**Table 1:**
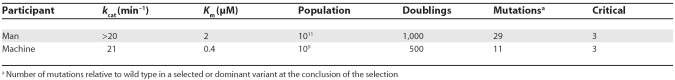
A Quantitative Comparison of Evolved Ribozymes

Why didn't the machine show a bigger win? It had the advantage of both pre-adaptation and stringent selection pressure (lowered substrate concentration). What were its disadvantages? Because the microfluidic platform used small volumes and because the rate of mutation was lower, the experiment started with a much smaller population, potentially limiting at least the initial diversity of variants. That said, when the effects of individual mutations on phenotype were examined via site-directed mutagenesis, only three mutations from each experiment were found to greatly influence the kinetic parameters of the selected ribozymes. More mutations accumulated from the initially larger, more diverse population, but not more important mutations.

So, really, the question is why didn't either experiment show a bigger win? Why didn't either ribozyme get much faster, since any speed (*k*_cat_) advantage would have resulted in a selective advantage (although potentially limited also by the rate of reverse transcription) [7]? It is taken as an article of faith that ribozymes are slower than proteins, and that this was one reason that they were displaced as the major catalytic machinery some 3 billion years ago. Indeed, Ron Breaker and his co-workers have suggested based on mechanistic considerations that ribozyme cleavases have a “speed limit” (1 min^−1^; [8]). This limit is similar to the top speed of the continuously evolved Bartel ligases, suggesting that these catalysts may have reached their limits, as well. If this is the case, it doesn't matter how fast Wright hammers or Paegel steams: the ribozyme itself can go no faster.

There is another possibility, though, for why faster ribozymes never evolved in the man–machine race, and it involves the rather unnatural birth of the ligase itself. Unlike natural sequences, which presumably have accumulated by the process of point mutation and recombination and thus have been highly constrained by the identity of their ancestors, the original Bartel ligase ribozyme was plucked from an unfettered sampling of an extremely large sequence space. In fact, when the information content of the ribozyme was determined it was found that it was something of a miracle: it should only have been selected once every 10,000 times or so that Bartel did the selection experiment. Now, this is commonly interpreted to mean not that Bartel was extremely lucky, but rather that there are many different (but equally rare) motifs that could have arisen during the selection process. If the “tape of evolution” [9] were to be run again, another such complex motif would come to the fore.

While this is undoubtedly true, it doesn't say anything about the nature of what did emerge; about the evolvability of the Bartel ligase. In fact, this molecule has been subjected to numerous different directed evolution experiments, and has in general proved extremely recalcitrant to sequence change [10]. Since this ribozyme was not born by the process of moving slowly through a sequence landscape, maybe it has little capability to evolve when pushed along a sequence landscape. It is possible that the Bartel ligase occupies a tall, lonely fitness peak, surrounded largely by deep, functionless valleys.

In order to access the additional catalytic mechanisms that Breaker and co-workers suggest would be necessary to break the “speed limit,” the Bartel ligase and other catalysts may need to move through relatively large sequence spaces (in contrast to at least some nucleic acid binding species which appear to crop up in even small sequence spaces) [11]. And unfortunately, this is exactly what has not occurred in continuous evolution experiments, either manual or automated. It may be that increased mutation rates will allow future continuous evolution experiments to traverse much more extensive sequence landscapes, and to find much faster or more chemically novel ribozymes. If so, while the machine will have the advantage in terms of turning rounds of selection, man will still have the edge in terms of manipulating the experimental variables to his liking.

Paegel and Joyce's advance will engender not additional tales of man versus machine, but of both synergizing with evolution. The man–machine interface is now poised to provide a truly quantitative picture of molecular evolution. It will be possible to routinely replicate selection experiments (something previously only attempted by only a few researchers, such as Niles Lehman), and to carry out multiple different selection experiments where key variables such as population size and stringency are systematically varied. The products of directed evolution may change qualitatively as well as quantitatively. Biologists are so used to thinking about evolution that we typically view it as a monolithic concept. However, while biological evolution does not usually admit to fully stopping either mode or tempo, directed evolution in the laboratory can occur discontinuously, a feature that limits speed but discourages the accumulation of parasites [12]. Machine-based continuous evolution should be the best of all worlds, combining man's mental ability to chart the future with automated control of selection stringency with the still unpredictable mode and tempo of evolution's relentless drive.
